# Antineutrophil Cytoplasmic Autoantibody-Associated Glomerulonephritis as a Possible Side Effect of COVID-19 Vaccination

**DOI:** 10.7759/cureus.30565

**Published:** 2022-10-21

**Authors:** Edva Noel, Urmiya Rashid, Rizwan Rabbani, Waqas Ahmad Khan, Yves-Smith Benjamin, Iris Lee

**Affiliations:** 1 Nephrology, Temple University Hospital, Philadelphia, USA; 2 Internal Medicine, Philadelphia College of Osteopathic Medicine (PCOM), Philadelphia, USA; 3 Nephrology, Riverside University Health System Medical Center, Moreno Valley, USA

**Keywords:** pr-3, mpo, nets, acute kidney injury, anca-associated glomerulonephritis, mrna sars-cov-2 vaccine

## Abstract

Vaccination is the principal tool aimed at curbing the COVID-19 pandemic that has, so far, affected tens of millions of individuals in the United States. The two available mRNA vaccines developed by Pfizer-BioNTech and Moderna possess high efficacy in preventing infection and illness severity. However, there are multiple side effects associated with these vaccines, some impacting different organs. Renal pathology is variable, with increasing cases of glomerulonephritis being observed. We report a rare acute kidney injury case due to antineutrophil cytoplasmic autoantibody (ANCA)-mediated glomerulonephritis after administering a second dose of the Pfizer-BioNTech mRNA SARS-CoV-2 vaccine. Aggravation and/or development of autoimmunity after mRNA vaccination may involve multiple immune mechanisms leading to de novo and recurrent glomerular diseases with an autoimmune basis.

## Introduction

SARS-CoV-2 has infected 62.4 million patients in the United States. With the emergence of new variants, this number is expected to grow. Vaccinating as many individuals as possible is critical to controlling the spread of disease. Approximately 75% of the American population has received at least one dose of vaccine [1]. Side effects, including fever, malaise, and injection site pain, are common, and most subside with analgesics and antipyretics. However, more severe side effects such as anaphylaxis and thrombosis can occur. Recently, a few cases of antineutrophil cytoplasmic autoantibody (ANCA)-associated glomerulonephritis triggered by the BNT162b2 mRNA SARS-CoV-2 vaccine were reported [2]. Herein, we summarize the clinical and laboratory characteristics of such cases based on a literature review and report a similar case of ANCA-associated glomerulonephritis, likely caused by BNT162b2.

## Case presentation

**Figure 1:**
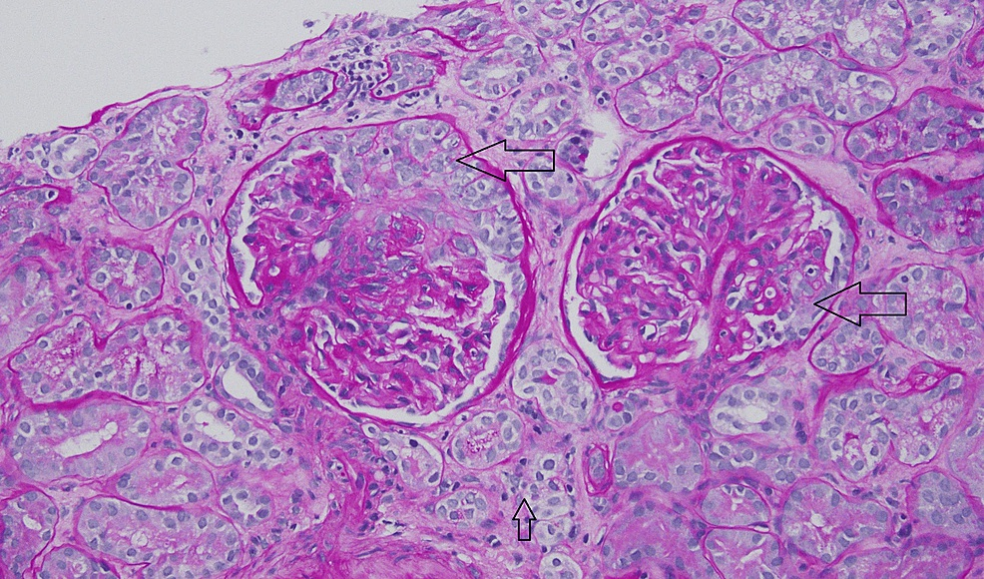
Kidney biopsy Periodic acid-Schiff stain showing two glomeruli with cellular crescent formation (big arrows), moderate-to-severe interstitial fibrosis, and tubular atrophy (small arrow).

A 62-year-old female with a history of limited systemic sclerosis complicated by pulmonary arterial hypertension, interstitial lung disease, and diabetes mellitus type 2 was admitted to the hospital for elevated serum creatinine of 5.18 mg/dL from a baseline of less than 1.0 mg/dL two months before presentation. The patient was asymptomatic except for increased urinary frequency, which she attributed to a recently increased dose of diuretics to manage worsening lower-extremity edema. The patient was on sildenafil 20 mg and macitetan 10 mg daily for pulmonary hypertension, dulaglutide, and pantoprazole 40 mg daily for gastroesophageal reflux disease due to esophageal dysmotility. She had received two doses of the Pfizer-BioNTech mRNA SARS-CoV-2 vaccine four and seven weeks prior to admission and reported no previous infection by SARS-CoV-2. Her blood pressure on admission was 150/90 mmHg. The rest of her vitals and physical examination were remarkable, only for a 2+ lower-extremity edema. Her acute kidney injury (AKI) did not resolve after stopping diuretics and administering normal saline. Renal ultrasound showed a normal kidney size and echotexture without hydronephrosis. Urine microscopy was abnormal, with 20 isomorphic red blood cells/high-power field (HPF). The urine protein-to-creatinine ratio by spot analysis was 0.8 g/g of creatinine. Complements were within the normal range except for a mildly low C3 at 80 mg/dL. Hepatitis B and C serologies were negative. The antinuclear antibody was positive with a titer of 1:640, and the perinuclear form of ANCA (P-ANCA) was positive with a titer of >1:640. Myeloperoxidase (MPO) antibody was elevated at 335.8 IU. A CT-guided kidney biopsy was performed, which showed crescentic glomerulonephritis with five out of 57 glomeruli manifesting cellular crescent formation. No endocapillary hypercellularity, fibrinoid necrosis, or segmental sclerosis was seen within the glomeruli. There was no arterial inflammation, necrosis, or thrombosis. Moderate-to-severe interstitial fibrosis and tubular atrophy were present. Immunofluorescence (IF) was negative. There was no evidence of a scleroderma renal crisis. (Figure 1). Hence, the diagnosis of P-ANCA-associated pauci-immune glomerulonephritis was made.

Due to the recent administration of the BNT162b2 mRNA (Pfizer/BioNTech) vaccine, it was thought that the vaccine triggered her P-ANCA-associated pauci-immune glomerulonephritis. The patient was treated with intravenous methylprednisolone 500 mg for three doses, followed by a tapering dose of oral prednisone and six cycles of cyclophosphamide 750 mg each. After completing the cyclophosphamide regimen, she was started on maintenance of mycophenolate 1 g twice daily and prednisone tapered to 5 mg daily. Eight months following her presentation, her serum creatinine improved to 2.6 mg/dL, the urine dipstick became negative for protein, and hematuria and the MPO antibody decreased to 12.0 IU.

## Discussion

Eleven billion doses of the coronavirus vaccine have been administered globally, and about 4 million are administered daily [2]. As the most extensive vaccination campaign in history is underway, adverse events from vaccination, including those affecting the kidneys, continue to be reported. In an article published in Kidney International, Rovin BH et al. were the first to coin the term COVID-19 vaccine-associated glomerular disease (CVAGD) and describe various kidney-related pathologies noted post BNT162b2 mRNA (Pfizer/BioNTech) and mRNA-1273 (Moderna) vaccinations. These included IgA nephropathy, minimal change disease, scleroderma renal crisis, membranous nephropathy, anti-glomerular basement membrane disease, IgG4-renal disease, and ANCA-associated vasculitis (AAV). These cases included both de novo and recurrent cases of glomerular disease [3]. In addition, the timing of disease onset varied, with some kidney disease entities like AAV manifesting much later after vaccination than others. The mechanisms that may account for observed differences in glomerular disease timing and manifestations have been proposed by several authors.

Bystander activation of the immune system, dysregulated immune responses, autoimmunity, and stimulation of specific T-cell subsets resulting in cellular immunity and damage have been considered drivers of disease after vaccination [3,4]. AAV is a necrotizing vasculitis affecting small vessels and is associated with ANCA specific for myeloperoxidase (MPO-ANCA) or proteinase-3 (PR3-ANCA). The major clinicopathologic variants include microscopic polyangiitis, granulomatosis with polyangiitis, and eosinophilic granulomatosis with polyangiitis. AAV can also occur in a single organ, a subset referred to as renal-limited AAV [5]. The global incidence and prevalence of AAV vary from 0.4 to 24 cases per million person-years and 300 to 421 cases per million population [6]. The involvement of the kidney in AAV varies from 54% to 97%, with 80% of patients developing glomerulonephritis within two years of disease onset [7]. It may also lead to rapidly progressive glomerulonephritis, characterized by crescents formation and clinical progression to end-stage kidney disease requiring dialysis. Drug-induced AAV can occur with hydralazine, allopurinol, levamisole, minocycline, and propylthiouracil. A handful of AAV cases have been temporally associated with influenza, hepatitis A and B, rubella, smallpox, tetanus, tuberculosis, and human papillomavirus vaccinations [8,9]. 

The mechanism of mRNA-vaccine-induced AAV is not fully understood. SARS-CoV-2 mRNA vaccine may drive a neutrophilic immune response because of bystander cytokine production. Cytokine-primed neutrophils and macrophages produce ANCA, causing the formation of neutrophil extracellular traps (NETs), which cause complement activation and endothelial dysfunction leading to AAV [10]. In addition, toll-like receptor ligation and cytokine production from dendritic cells and macrophages recognizing components of mRNA further activate neutrophils and the production of NETs [11]. NETs are highly pro-inflammatory and provide a sustained antigenic stimulus. As part of innate immunity, NETs formation is essential for host defense. However, decreased clearance of NETs, prolonged exposure, and activation in immunocompromised individuals and high inflammatory states, as seen in infections like SARS-CoV-2 or vaccination like the mRNA vaccine, can disrupt tolerance leading to autoimmunity. Defects in NETs clearance in systemic lupus erythematosus (SLE) are a driver of disease and may explain renal disease flares observed in some SLE patients. MPO and PR-3, integral components of NETs, are usually intracellular and, once exposed to the immune system, serve as an antigenic stimulus favoring the formation of autoantibodies to MPO and PR-3 [12,13].

Both antigen-specific and nonspecific mechanisms could cause vaccine-associated autoimmunity. Antigen-specific causes of vaccine-associated autoimmunity may arise from molecular mimicry. SARS-CoV-2 spike glycoproteins may have homology to specific human proteins leading to cross-reactivity, immune responses to self-antigens, and the development of autoantibodies, especially in people with “immunological and serological” predispositions [14]. Other major nonspecific mechanisms in response to the vaccine include bystander immune activation, an inflammatory environment that elicits exposure of self-antigens previously shielded from the immune system, and excessive activation of innate immunity, which may contribute to the development of glomerular disease. Cases of ANCA-associated glomerulonephritis secondary to SARS-CoV-2 infection have been published and share similar mechanisms as vaccine-induced ANCA glomerulonephritis. In either case, NETs formation seems to be the strongest of all the proposed mechanisms. 

Three published cases of ANCA glomerulonephritis following COVID-19 vaccination share similarities with our case [15-17]. The presenting symptoms vary from asymptomatic to central nervous system symptoms (headache), gastrointestinal symptoms (nausea, vomiting, diarrhea), and constitutional symptoms (fever, malaise). Renal biopsies in all three cases showed crescentic fibrinoid glomerulonephritis with a negative IF (Table 1). All cases were managed with immunosuppressive therapy, differing in some cases based on the specific agent used (steroids and cyclophosphamide or rituximab). Our patient had a preexisting autoimmune condition, but others did not. Our patient had no clinical features of vasculitis on presentation, had AKI, and did not require dialysis. She had a de novo glomerulonephritis with crescents, but no other severe features, such as fibrinoid necrosis, were observed. Our patient was treated with cyclophosphamide and then continued maintenance therapy with mycophenolate on the basis that these medications are also effective in treating scleroderma. Eight months after presentation, our patient achieved remission with no evidence of proteinuria, hematuria, or ANCA and a serum creatinine that stabilized at 2.5 mg/dL.

**Table 1:** Characteristics of ANCA-associated glomerulonephritis after administration of mRNA CoVID-19 vaccines ANA: antinuclear antibody; F: female; M: male; sCr: serum creatinine; UA: urinalysis; UPCR: urine protein-to-creatinine ratio; RBC: red blood cell; WBC: white blood cell; EM: electron microscopy; IF: immunofluorescence; RTX: rituximab; CYC: cyclophosphamide; MMF: mycophenolate; MPO: myeloperoxidase antibody; PR-3: proteinase-3 antibody; ANCA: antineutrophil cytoplasmic autoantibody.

	Age/sex	Vaccine	Symptoms	Timing of onset	Peak sCr (mg/dL)	UA	UPCR (g/g)	Urine sediment	Complement	ANCA	ANA	Pathology	Treatment	Outcome
Shakoor et al. [15]	78 F	Pfizer-BioNTech COVID-19 vaccine	Nausea, vomiting, diarrhea, lethargy	28 days after first dose	3.54	3+ RBC, 2+ protein	Not reported	Dysmorphic RBC, few WBC	Normal levels	MPO+	Negative	LM - crescentic necrotizing glomerulonephritis. IF - negative, EM - not reported	Steroid and RTX	Serum creatinine improved; no dialysis required
Sekar et al. [16]	52 M	Moderna (mRNA-1273)	Headache and weakness	2 weeks after second dose	10.42	1+ protein	Not reported	Dysmorphic RBC	C3 and C4 normal	PR3+ MPO-	Not reported	LM - crescent and fibrinoid necrosis. IF - negative, EM - segmental fibrin staining	Pulse steroids and RTX were replaced by CYC due to intolerance to RTX	Continued hemodialysis
Obata et al. [17]	84 M	BNT 162b2 (Pfizer/BioNTech)	Fever, malaise, cough	2 weeks after second dose	1.22	3+ RBC, 1+ protein	0.19	Not reported	Not reported	MPO+	Not reported	LM - focal necrotizing glomerulonephritis with cellular crescents. IF - negative. EM - not reported	High-dose methylprednisolone followed by oral prednisone	Serum creatinine 1.35 from 1.22 eight weeks after presentation; no dialysis required
Our case	62 F	BNT 162b2 (Pfizer/BioNTech)	Lower extremities edema	4 weeks after the second dose	5.18	3+ RBC 1+ protein	0.8	Dysmorphic RBC, no RBC casts	Mildly low C3, normal C4	MPO+	Positive	LM - crescentic glomerulonephritis. IF - negative. EM - not available	High-dose methylprednisolone followed by oral prednisone + 6 doses of monthly CYC 720 mg followed by maintenance MMF 1 g bid	Serum creatinine improved from 6.70 to 2.5 eight months after presentation; no dialysis was required

## Conclusions

As increased cases of AAV following the administration of the COVID-19 vaccine are reported, healthcare providers should be aware of the diverse adverse effects that are increasingly reported in association with mRNA vaccines. Among those are the CVAGDs like the one reported in this case. At present, the implication of causation remains at the level of these four case reports, but further studies are needed to substantiate the evidence. For nephrologists, particular attention must be paid to patients with autoimmune conditions and those with a previous history of glomerulonephritis after receiving the COVID-19 vaccine. Since CVAGDs respond well to immunosuppressive treatments, early detection may lead to a favorable outcome.
